# Lower Drug Survival, Less Satisfaction and More Adverse Events in Females Using Biologics for Psoriasis: Results of the Dutch BioCAPTURE Registry

**DOI:** 10.1177/24755303251327926

**Published:** 2025-04-01

**Authors:** Liana Barenbrug, Renate G. van der Molen, Jake S. F. Maurits, Marieke M. B. Seyger, Marisol E. Otero, Antoni H. Gostynski, Femke M. Homan, Paul M. Ossenkoppele, Inge M. Haeck, Judith. H. J. Hendricksen-Roelofzen, John E. M. Körver, Sharon R. P. Dodemont, Berit Velstra, Maartje A. M. Berends, Lizelotte J. M. T. Weppner-Parren, Romy Keijsers, Annet M. Oostveen, Bas Peters, Roland J. M. Mommers, Martijn B. A. van Doorn, Milan Tjioe, Peter W. Arnold, Astrid L. A. Kuijpers, Marloes M. Kleinpenning, Elke M. G. J. de Jong, Juul M. P. A. van den Reek

**Affiliations:** 1 Department of Dermatology, 6034 Radboud University Medical Center (Radboudumc), Nijmegen, The Netherlands; 2 Department of Laboratory Medicine, Laboratory of Medical Immunology, 6034 Radboud University Medical Center (Radboudumc), Nijmegen, The Netherlands; 3 IQ Health Science Department, 6034 Radboud University Medical Center (Radboudumc), Nijmegen, The Netherlands; 4 Department of Dermatology, 199236 Maastricht University Medical Center+, Maastricht, The Netherlands; 5 Department of Dermatology, 1154 Ziekenhuisgroep Twente, Almelo/Hengelo, The Netherlands; 6 Department of Dermatology, 8124 Utrecht Medisch Centrum, Utrecht, The Netherlands; 7 Department of Dermatology, 36814 Streekziekenhuis Koningin Beatrix, Winterswijk, The Netherlands; 8 Department of Dermatology, 89411 Amphia Ziekenhuis, Breda, The Netherlands; 9 Department of Dermatology, 3168 Catharina Ziekenhuis, Eindhoven, The Netherlands; 10 Department of Dermatology, 6028 St Antonius Ziekenhuis, Nieuwegein, The Netherlands; 11 Department of Dermatology, 2987 Slingeland Ziekenhuis, Doetinchem, The Netherlands; 12 Department of Dermatology, 10233 Jeroen Bosch Ziekenhuis, ’s Hertogenbosch, The Netherlands; 13 Department of Dermatology, 159205 Zuyderland Medisch Centrum, Sittard-Geleen/Heerlen, The Netherlands; 14 Department of Dermatology, 72485 Gelre Ziekenhuizen, Apeldoorn, The Netherlands; 15 Department of Dermatology, 1322 Ziekenhuis Rijnstate, Arnhem, The Netherlands; 16 Department of Dermatology, 61178 Anna Ziekenhuis, Geldrop, The Netherlands; 17 Department of Dermatology, 6993 Erasmus MC, Rotterdam, The Netherlands; 18 Department of Dermatology, Bravis, Bergen op Zoom, The Netherlands; 19 Department of Dermatology, 3096 Ziekenhuis Gelderse Vallei, Ede, The Netherlands; 20 Department of Dermatology, 89569 Máxima MC, Eindhoven, The Netherlands; 21 Department of Dermatology, 6030 Canisius Wilhelmina Ziekenhuis, Nijmegen, The Netherlands

**Keywords:** psoriasis, sex-differences, biologics, IL17 inhibitor, IL23 inhibitor

## Abstract

**Background:**

Drug survival of biologics for psoriasis has reported to be lower in females than males for first-generation biologics (TNF-α/interleukin (IL) 12/23 inhibitors (i)); insights for newer biologics (IL17i and IL23i) are scarce.

**Objectives:**

To study sex-differences in drug survival and other treatment outcomes of biologics (including IL17i/IL23i) in patients with psoriasis.

**Methods:**

Data were obtained from the Dutch, prospective, multicenter, BioCAPTURE registry. Kaplan-Meier drug survival curves were split for specific discontinuation reasons and stratified for sex. Cox regression models with confounder correction were used to investigate the association of sex with drug survival. Adverse events (AEs) leading to biologic discontinuation were compared between sexes. Confounder-corrected Generalized Estimated Equation models were used to compare the course Psoriasis Area and Severity Index (PASI), Treatment Satisfaction Questionnaire for Medication (TSQM)) scores, and Dermatology Life Quality Index (DLQI) scores between sexes.

**Results:**

We included 428 females and 703 males (respectively 744 and 1069 treatment episodes). For all biologics, female sex was associated with shorter overall, AE-related, and effectiveness-related drug survival. For IL17i/IL23i specifically, female sex was associated with shorter overall and effectiveness-related drug survival, but not with shorter AE-related drug survival. In the TSQM females reported to experience more often AEs and to be, in general, less satisfied than males. No sex-differences were found for PASI and DLQI during the first year of treatment.

**Conclusion:**

Biologics, including IL17i and IL23i, showed lower drug survival rates for females. This could be linked to the sex-differences we found regarding AEs and treatment satisfaction with biologics**.**

## Graphical abstract

**Graphical Abstract:**
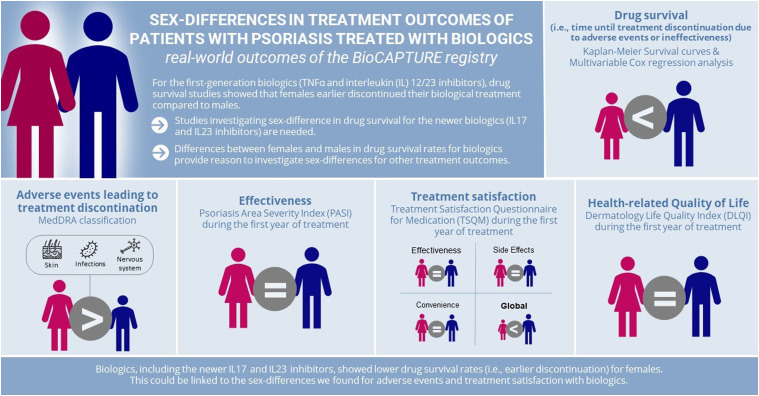

## Introduction

Psoriasis is a chronic inflammatory skin disease affecting 2%–3% of the Western population.^1^ Biologics are an effective treatment for psoriasis. The performance of biologics in real-world practice can be measured in terms of the time patients remain on a prescribed drug, using drug survival analysis.^2^ Previously, female sex was repeatedly associated with earlier discontinuation of biologics compared to male sex in multiple drug survival studies.^3-6^ The underlying mechanism behind this observation is not fully elucidated, though sex-differences are present with regards to psoriasis in general. Despite equal prevalence of psoriasis in both sexes, males more often have more severe psoriasis at baseline.^7-9^ Contrastingly, females reported a lower health-related quality of life (HR-QoL) due to their psoriasis compared to males, and had higher treatment expectations.^10-17^ Also, females with psoriasis have an increased risk of cardiovascular, metabolic or psychological comorbidities, which might influence the tolerability of used drugs.^18-21^

However, most studies were not focused on biologics resulting in limited insight on the reasons behind the observation of lower drug survival of biologics for females vs males. In addition, the newer interleukin (IL) 17 and IL23 inhibitors (i) have been understudied in the context of sex-differences and drug survival.

Therefore, the aim of this study is to assess differences in drug survival between females and males in first- and second-generation biologics, and possible sex-differences in treatment outcomes such as disease activity, adverse events (AEs), treatment satisfaction and HR-QoL within the prospective real-world BioCAPTURE cohort.

## Patients and Methods

For this explorative study data were extracted from the prospective, multicenter, long-term Continuous Assessment of Psoriasis Use Registry with Biologics (BioCAPTURE; https://www.biocapture.nl/). Since 2005, data from patients with psoriasis treated with biologics were registered in the database currently including data from 4 academic and 19 non-academic centers in the Netherlands. BioCAPTURE was approved by the ethics committee. Although not mandatory for this non-interventional study according to Dutch Law, informed consent was obtained from every patient in this registry. This study was reported according to the Strengthening the Reporting of Observational Studies in Epidemiology (STROBE) guidelines.^22^

### Data Collection

Data were extracted in April 2023 (data lock) for the following biologics: Tumor Necrosis Factor (TNF) α inhibitors (infliximab, etanercept, adalimumab), IL12/23i ustekinumab, IL17i (secukinumab, ixekizumab, brodalumab, bimekizumab) and IL23i (guselkumab, risankizumab, tildrakizumab). Certolizumab pegol was excluded from this study because it is often prescribed in pregnant women for safety reasons. Baseline characteristics (sex, age at onset of psoriasis, disease duration until start of biological therapy, age at start of biological therapy, biologic class and type, body mass index (BMI), Psoriasis Area and Severity Index (PASI) scores, presence of psoriatic arthritis (PsA), family history with psoriasis, experience with prior biologics, previous conventional systemic medications, comorbidities), AEs leading to treatment discontinuation and questionnaires filled in by patients (Treatment Satisfaction Questionnaire for Medication (TSQM) and Dermatology Life Quality Index (DLQI)) were extracted. Biological sex was extracted as how it was reported in electronic patient records.

### Drug Survival

A treatment episode was defined as a continuous period of time in which a patient was treated with a certain biologic type, with no interruption >90 days. If a patient received different types of biologics in our registry, this patient has a treatment episode for each biologic type (i.e., one patient can have multiple treatment episodes). If a patient received >1 treatment episode of the same biologic type in our registry, only the first treatment episode of that biologic type was analyzed. We analyzed drug survival rates using Kaplan–Meier estimates. For the overall drug survival curve all reasons of discontinuation were considered an event, such as ineffectiveness, AEs, death, and pregnancy. Patients were censored when lost to follow-up, or if still using the biologic at data lock. Additionally, we assessed drug survival separately for ineffectiveness and AEs. For these sub-analyses, patients were censored when they discontinued their biologic for a reason other than the reason of interest.

Multivariable Cox regression models, including possible confounding factors based on clinical relevance (age at start of biological therapy, duration of psoriasis until start of biological therapy, baseline PASI, BMI, PsA, cardiovascular disease, diabetes mellitus, inflammatory bowel disease or depression, biologic type, experience with prior biologics) were used to identify the association of sex with drug survival for (1) all biologics and (2) IL17i and IL23i together. All confounding factors were assessed at baseline (i.e., the start of the treatment episode of interest). PsA, cardiovascular disease, diabetes, inflammatory bowel disease and depression were defined as comorbidities, when the onset of these conditions was before the initiation of the treatment episode.

### Adverse Events Leading to Treatment Discontinuation

All AEs that led to discontinuation of a biologic were collected and classified into categories according to the Medical Dictionary for Regulatory Activities (MedDRA). If patients had >1 AE simultaneously leading to treatment discontinuation, these were counted as separate AEs.

### Psoriasis Area and Severity Index (PASI)

To be able to visualize treatment effectiveness, PASI scores were analyzed for all biologics together. For each treatment episode, a PASI score was selected for 3, 6, 9 and 12 months with the assessment date closest to the respective time point. Linear interpolation was used to estimate missing PASI scores, when a baseline PASI and >1 follow-up PASI within the first year of treatment was available. When one or more of these time points could not be estimated using linear interpolation, multiple imputation was applied. Imputed variables were created and pooled in the model 10 times. Additionally, PASI scores at the time of treatment discontinuation due to ineffectiveness were assessed. We checked potential differences between the population with and without baseline PASI.

### Treatment Satisfaction Questionnaire for Medication (TSQM) and Dermatology Life Quality Index (DLQI)

Patients in the registry were asked to fill in TSQM-vII (Appendix S1) and DLQI questionnaires at baseline and at 3, 6, 9 and 12 months of treatment. The TSQM is a generic validated questionnaire covering patients’ satisfaction of four domains: effectiveness, convenience, global satisfaction and side-effects.^23^ Every domain ranges from zero (extremely dissatisfied) to 100 (extremely satisfied). The DLQI is a dermatology-specific, validated questionnaire consisting of 10 question concerning HR-QoL, generating score ranging from zero (no impairment) to 30 (maximum impairment).^24^ For the time points where scores were missing, multiple imputation was applied. Imputed variables were created and pooled in the model 10 times. The number of treatment episodes included in the TSQM and DLQI analyses is lower than the total study population, as only treatment episodes in which a TSQM or DLQI was filled in at least once during the first year of treatment could be included in the analysis. We checked potential differences between the population with and without TSQM/DLQI scores.

### Statistical Analysis

All analyses were performed in SPSS version 29.0 (IBM Corporation, Armonk, NY, USA). A *P*-value <0.05 was considered significant. Baseline patient and treatment characteristics were displayed using descriptive statistics (mean ± standard deviation (SD)), median [interquartile range (IQR)], N (%)). Continuous variables were compared between patient groups using an unpaired *t* test for normal distributions and Mann–Whitney U tests for skewed distributions. Pearson’s Chi-square test was used for binary variables.

The effects of sex on the course of PASI, TSQM and DLQI during the first year of treatment was analyzed using Generalized Estimated Equations (GEE), to take into account the different number of treatment episodes per patient and the longitudinal measurements. A model was built for each outcome measure (PASI, TSQM subdomains, DLQI), where the outcome measure was defined as dependent, and sex as independent variable. For each model the same possible confounding factors as used in the multivariable Cox-regression model were incorporated plus the baseline score of the dependent variable of interest. Model assumptions were checked with residual plots.

## Results

### Patient Characteristics

This study included 428 females and 703 males, with respectively 744 and 1069 treatment episodes. The total observation years were 5066.38, and for females and males respectively 1829.44 and 3236.94. Most baseline characteristics were comparable for females and males (Table 1, baseline characteristics per biologic type see Table S1/S2). Baseline PASI was lower in females (9.9 [9.6] vs 11.1 [8.8], *P* = 0.013) and slightly more females had concomitant PsA (33.2% vs 28.7%, *P* = 0.049). Also, the type of systemics used before biologic initiation was different between sexes, with more ciclosporin and less retinoids for females versus males.

**Table 1.** Patient and Treatment Characteristics of the First Treatment Episode in BioCAPTURE of Females and Males.

	Total	Females	Males	P
Number of patients (n (%))	1131 (100%)	428 (37.8%)	703 (62.2%)	
Age at onset psoriasis, years (median [IQR])^a^	23.5 [20.0]	21.9 [23.0]	24.0 [19.0]	0.059
Disease duration until start of biological therapy, years (median [IQR])^b^	18.8 [17.4]	18.4 [18.6]	19.3 [16.7]	0.309
Age at start of biological therapy, years (mean (SD))^c^	48.8 (13.6)	48.4 (14.5)	49.1 (13.0)	0.419
Baseline BMI, kg/m2 (median [IQR])^d^	27.9 [7.0]	28.3 [8.3]	27.8 [5.3]	0.440
Baseline PASI score (median [IQR])^e^	10.7 [9.2]	9.9 [9.6]	11.1 [8.8]	**0.013**
PsA, yes^f^	289 (30.5%)	122 (33.2%)	167 (28.7%)	**0.049**
Family history with psoriasis, yes^g^	664 (64.9%)	263 (66.9%)	401 (63.7%)	0.166
Biologic naïve, yes^h^	748 (68.4%)	249 (67.4%)	469 (69.0%)	0.586
Number of previous used conventional systemic medication before start of biologic (median [IQR])	2.0 [1.0]	2.0 [1.0]	2.0 [1.0]	0.861
Type of previous conventional systemics before start biologic				
Ciclosporin	275 (14.2%)	117 (16.3%)	158 (13%)	**0.043**
Fumaric acid	507 (26.3%)	184 (25.6%)	323 (26.6%)	0.669
Methotrexate	880 (45.6%)	332 (46.4%)	548 (45.1%)	0.590
Systemic retinoids	261 (3.5%)	77 (10.8%)	184 (15.1%)	**0.006**

Missing data;

^a^n = 86 (7.6%),

^b^n = 79 (7.0%),

^c^n = 4 (0.4%),

^d^n = 231 (20.4%),

^e^n = 172 (15.2%),

^f^n = 183 (16.2%),

^g^n = 106 (9.4%),

^h^n = 37 (3.3%).

Percentages are calculated using the total number of available data for that characteristic per group.

IQR, interquartile range; SD, standard deviation; BMI, body mass index; PASI, Psoriasis Area and Severity Index; PsA, psoriatic arthritis.

### Drug Survival

**Figure 1.**
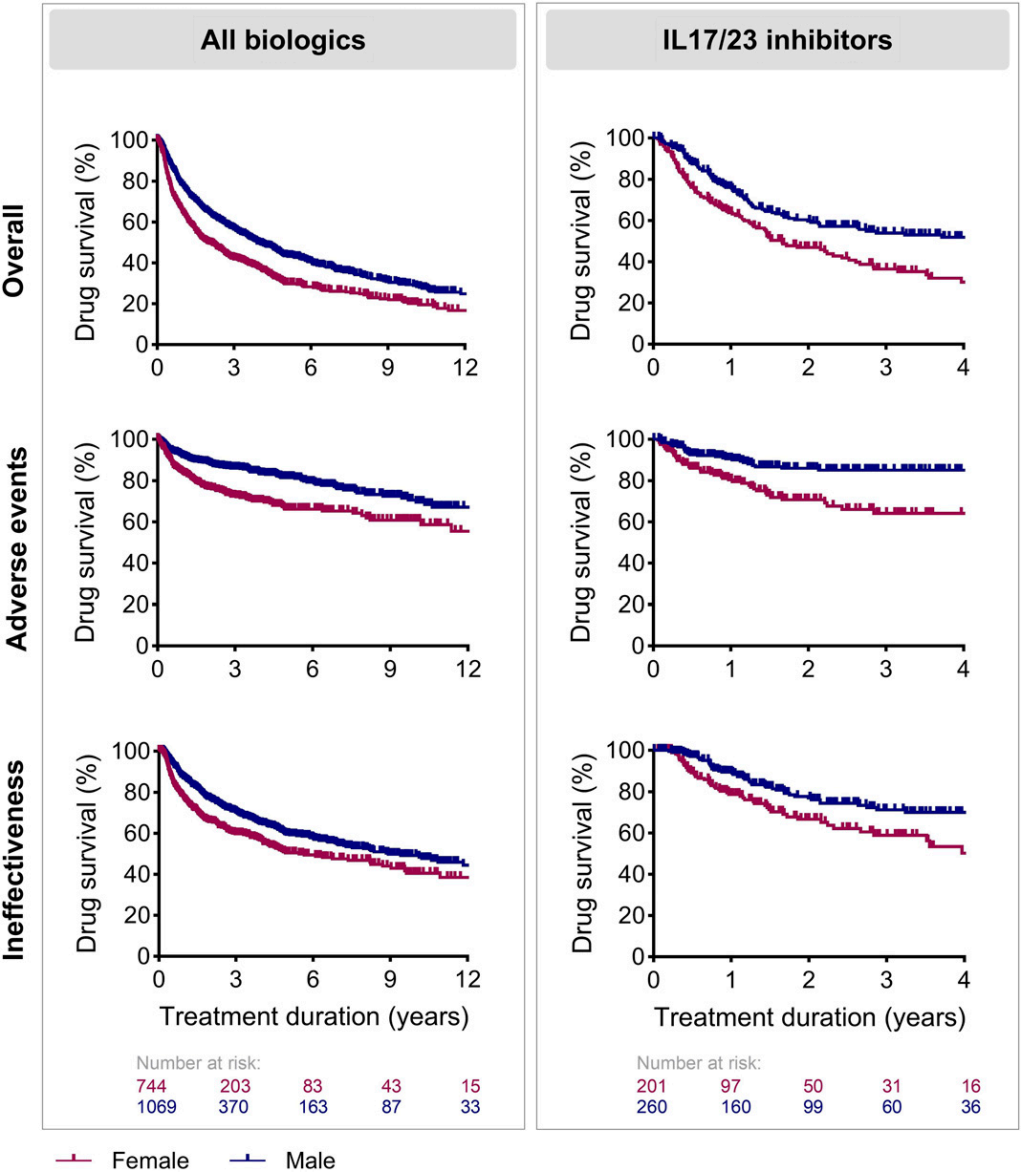
Drug survival of biological therapy in females compared to males, split according to reason of discontinuation. All biologics or all IL17 and IL23 inhibitors were included in Kaplan-Meier analysis.

Raw drug survival curves for all biologics together and for the IL17i/IL23i group, showed a shorter drug survival for females compared to males (Figure 1). The 3-year overall drug survival for all biologics was 42.1% and 56.6% for females and males respectively. For the IL17i/IL23i group, the 3-year overall drug survival was 36.5% for females and 53.9% for males. Raw drug survival curves of each IL17i and IL23i separately suggest that drug survival was also shorter for females versus males for secukinumab and risankizumab (Figure S1). Multivariable Cox regression analyses were used to show the association with sex and treatment discontinuation, corrected for confounders. For all biologics combined, female sex was associated with shorter overall drug survival (Hazard ratio (HR) = 1.535 [95% confidence interval (CI) 1.278-1.844], *P* < 0.001)), drug survival related to AEs (HR = 1.939 [95%CI 1.404-2.677], *P* < 0.001), and drug survival related to ineffectiveness (HR = 1.499 [95%CI 1.180-1.906], *P* < 0.001). For IL17i/IL23i, female sex was associated with shorter overall drug survival (HR = 2.126 [95%CI 1.243-3.636], *P* = 0.006) and drug survival related to ineffectiveness (HR = 2.808 [95%CI 1.366-5.770], *P* = 0.005). Although a trend for a shorter drug survival in females was seen for drug survival related to AEs in Kaplan-Meier curves, no statistically significant association was found for the IL17i/IL23i subgroup (HR = 2.479 [96%CI = 0.944-6.507], *P* = 0.065).

### Adverse Events Leading to Treatment Discontinuation

In total, 316 (17%) treatment episodes were discontinued due to one or more AEs (Table 2). In the total group, infections and infestations, musculoskeletal disorders, skin and subcutaneous disorders and general disorders were the most common AEs. The most obvious sex-discrepancy in the proportion of AEs leading to treatment discontinuation per group were recorded for gastrointestinal disorders (females; 2.3% vs males; 0.8%), nervous system disorders (females; 2.8% vs males; 1.0%), skin and subcutaneous disorders (females; 3.0% vs males; 1.2%), and infections and infestations (females; 8.1% vs males; 3.8%).

**Table 2.** Adverse Events Leading to Treatment Discontinuation of a Biologic in Females Compared to Males.

Adverse Events (MedDRA Classification)	Total N TEs = 1813	Female N TEs = 744	Male N TEs = 1069
Blood and lymphatic system disorders	1 (0.06%)	0 (0%)	1 (0.09%)
Cardiac disorders	8 (0.44%)	3 (0.40%)	5 (0.47%)
Ear and labyrinth disorders	1 (0.06%)	0 (0%)	1 (0.09%)
Endocrine disorders	2 (0.11%)	1 (0.13%)	1 (0.09%)
Eye disorders	3 (0.17%)	2 (0.27%)	1 (0.09%)
Gastrointestinal disorders	26 (1.43%)	**17 (2.28%)**	**9 (0.84%)**
General disorders and administration site conditions	54 (2.98%)	**31 (4.17%)**	**23 (2.15%)**
Allergic reactions	8 (0.44%)	4 (0.54%)	4 (0.37%)
Injection site reactions	8 (0.44%)	5 (0.67%)	3 (0.28%)
Fatigue	18 (0.99%)	9 (1.21%)	9 (0.84%)
Fever	4 (0.22%)	1 (0.13%)	3 (0.28%)
Oedema	4 (0.22%)	3 (0.40%)	1 (0.09%)
Malaise	7 (0.39%)	4 (0.54%)	3 (0.28%)
Other^a^	4 (0.22%)	4 (0.54%)	0 (0%)
Hepatobiliary disorders	2 (0.11%)	1 (0.13%)	1 (0.09%)
Immune system disorders	7 (0.39%)	5 (0.67%)	2 (0.19%)
Infections and infestations	101 (5.57%)	**60 (8.06%)**	**41 (3.83%)**
Upper respiratory infections/flu-like symptoms	29 (1.59%)	16 (2.15%)	13 (1.22%)
Pneumonia	10 (0.55%)	3 (0.40%)	7 (0.65%)
Skin infections	13 (0.71%)	9 (1.21%)	4 (0.37%)
Urinary tract infections	9 (0.50%)	8 (1.08%)	1 (0.09%)
Sepsis	4 (0.22%)	2 (0.27%)	2 (0.19%)
Other^b^	34 (1.88%)	22 (2.96%)	12 (1.12%)
Investigations	8 (0.44%)	3 (0.40%)	5 (0.47%)
Metabolism and nutrition	1 (0.06%)	1 (0.13%)	0 (0%)
Musculoskeletal and connective tissue disorders	64 (3.53%)	28 (3.76%)	36 (3.37%)
Neoplasms benign, malignant, and unspecified	25 (1.38%)	12 (1.61%)	13 (1.22%)
Nervous system disorders	32 (1.77%)	**21 (2.82%)**	**11 (1.03%)**
Psychiatric disorders	16 (0.88%)	**9 (1.21%)**	**7 (0.65%)**
Renal urinary disorders	1 (0.06%)	1 (0.13%)	0 (0%)
Respiratory, thoracic, and mediastinal disorders	15 (0.83%)	**9 (1.21%)**	**6 (0.56%)**
Skin and subcutaneous tissue disorders	35 (1.93%)	**22 (2.96%)**	**13 (1.22%)**
Social circumstances	1 (0.06%)	0 (0%)	1 (0.09%)
Surgical and medical procedures	15 (0.83%)	**9 (1.21%)**	**6 (0.56%)**
Vascular disorders	7 (0.39%)	5 (0.67%)	2 (0.19%)
Unknown	1 (0.06%)	1 (0.13%)	0 (0%)
Total number of AEs leading to treatment discontinuation	426	241	185
Total number of TEs discontinued due to AEs	316 (17.43%)	168 (22.58%)	148 (13.84%)

Data expressed as n (%).

Percentages are calculated using the total number of treatment episodes per group.

Ninety-four patients (54 females and 40 males) had more than one adverse event leading to treatment discontinuation.

For the MedDRA classification category product issues, congenital, familial and genetic disorders; injury, poisoning and procedural complications; reproductive system and breast disorders; and product issues, there were no adverse events that led to treatment discontinuation were reported.

MedDRA; Medical Dictionary for Regulatory Activities.

^a^Pain on the chest after biologic injection, poor appetite, night sweats.

^b^Abscess, latent tuberculosis infection, recurrent infections, toe infection, infection in gastro-intestinal tract, ear infection, eye infection.

### Disease Activity

**Figure 2.**
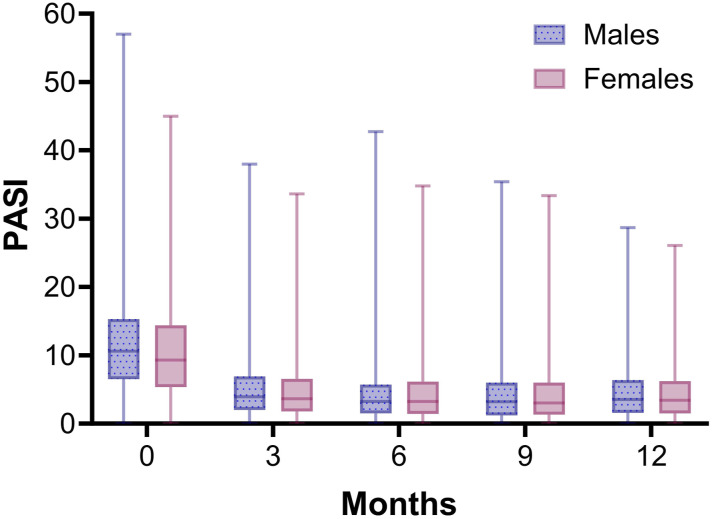
Median PASI scores for male and female patients over 1 year of treatment with a biologic. All biologics were included in the analysis. N = 879 and N = 601 treatment episodes for respectively males and females. Whiskers show minimum and maximum PASI scores.

Median baseline PASI differed between females and males (respectively 9.9 [IQR = 9.6] and 11.1 [IQR = 8.8], *P* = 0.013). Longitudinal analysis in both sexes showed that PASI scores rapidly decreased during the first 3 months of treatment with a biologic, and then stabilized (Figure 2). The corrected GEE model showed that the course of PASI during the first year of treatment with a biologic was comparable between females and males (Table S3). In patients discontinuing treatment due to ineffectiveness median PASI at discontinuation was 7.8 [IQR = 7.8] for females compared to 9.1 [IQR = 8.5] for males (*P* = 0.083).

### Treatment Satisfaction

**Figure 3.**
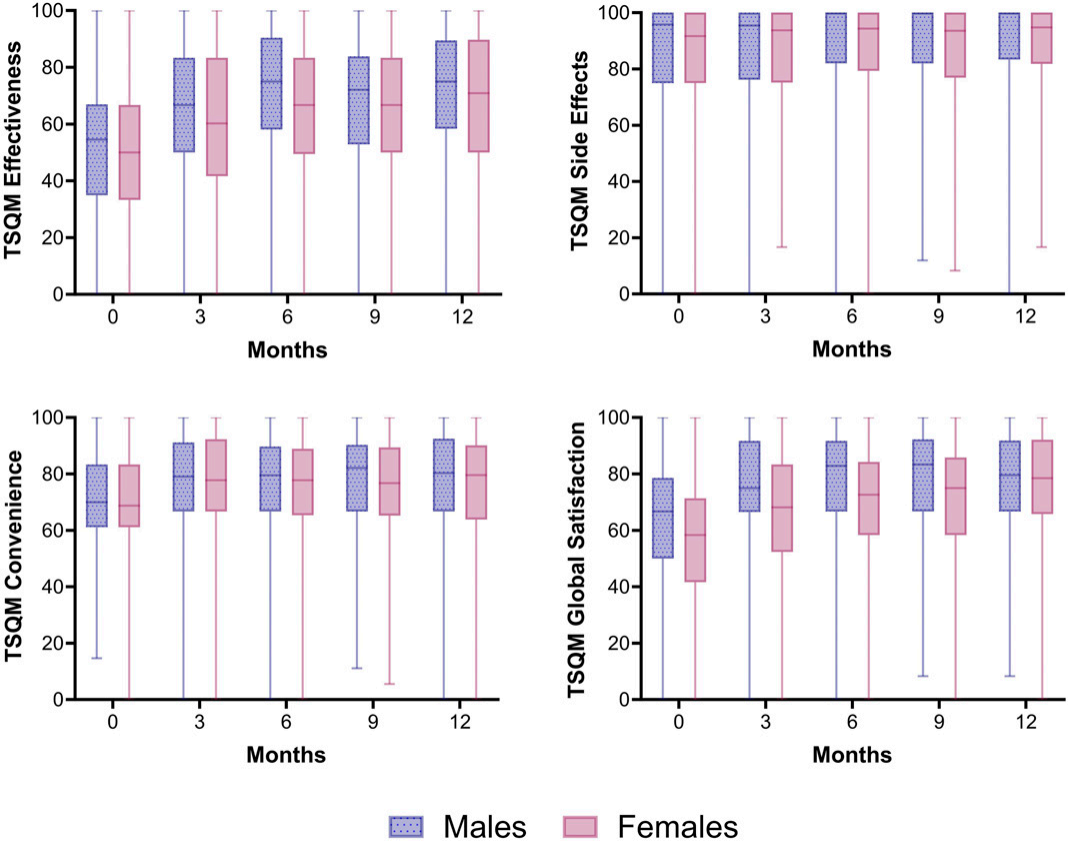
Treatment satisfaction of biologics in male and female patients with psoriasis. Median TSQM scores for each domain (effectiveness, side effects, convenience, and global satisfaction) for female and male patients over 1 year of treatment with a biologic. All biologics were included in the analysis. N = 510 and N = 398 treatment episodes for respectively males and females. Whiskers show minimum and maximum TSQM scores.

**Figure 4.**
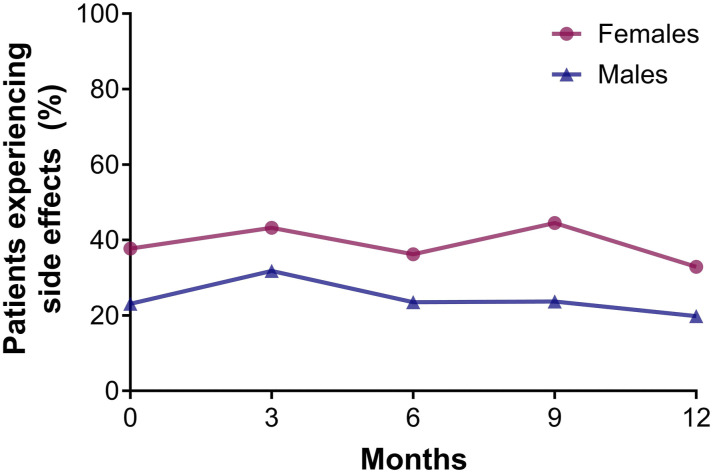
Percentage of male and female patients reporting any side effects as a consequence of biological therapy, as reported in the TSQM. All biologics were included in the analysis. N = 510 and N = 398 treatment episodes for respectively males and females.

Females were slightly less satisfied with their biologic treatment than males (Figure 3). Using GEE corrected for confounders no significant sex-differences were found regarding satisfaction with treatment effectiveness (domain Effectiveness), safety profile (domain Side Effects) and the practical use of the biologic (domain Convenience) (Table S4-6). General satisfaction (domain Global Satisfaction) with biologics was statistically significantly higher for males than females (2.694, [95%CI 0.515-4.873], *P* = 0.015) (Table S7). Females more often reported experiencing side effects from the biologic than males during the first treatment year (i.e., at 1 year 33.0% vs 19.8%) (Figure 4).

### HR-QoL

**Figure 5.**
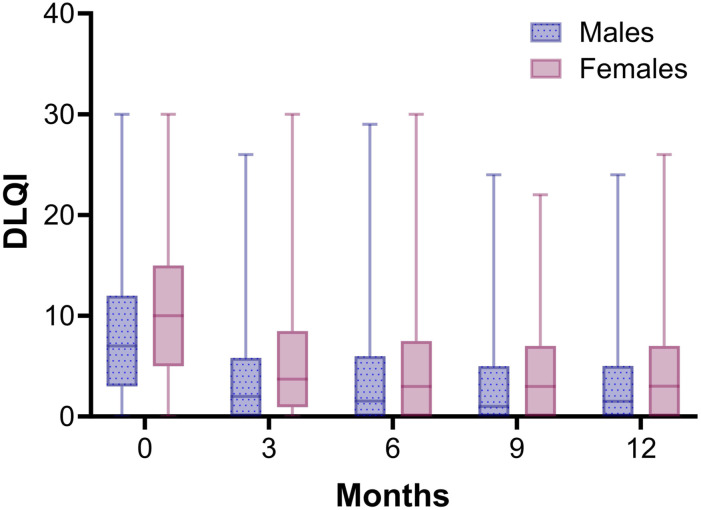
Median DLQI scores for male and female patients over 1 year of treatment with a biologic. All biologics were included in the analysis. N = 547 and N = 418 treatment episodes for respectively males and females. Whiskers show minimum and maximum DLQI scores.

The first 3 months upon initiation of a biologic the median DLQI score decreased and then stabilized for both females and males (Figure 5). Also after correction for confounders, the GEE model showed that the course of DLQI during the first year of treatment with a biologic was comparable between females and males (Table S8).

No difference in patient characteristics were found comparing the group of patients with missing TSQM/DLQI/baseline PASI versus patients with TSQM/DLQI/baseline PASI score.

## Discussion

This study investigated sex-differences in treatment outcomes of patients with psoriasis treated with biologics using real-world data. Female sex was associated with shorter overall drug survival for all biologics combined and with AE and ineffectiveness related drug survival. For the IL17i/IL23i group, female sex was associated with shorter overall drug survival and shorter ineffectiveness related drug survival, but not with shorter AE related drug survival. In the TSQM females reported more often to experience AEs and to be in general less satisfied with their biologic in the first year of treatment compared to males. There was no sex-difference in disease activity and HR-QoL during the first year of treatment with a biologic.

Female sex was previously reported as predictor for discontinuation of older biologics, but studies on the newer IL17i and IL23i are scarce.^3-5^ One study on sex-differences in drug survival of IL17i showed lower survival rates for females for secukinumab and ixekizumab, however, reasons of discontinuation were unknown.^6^ Our study affirms that drug survival of biologics, including the IL17i/IL23i, was lower for females compared to males. Survival curves showed prominent sex-differences for secukinumab and risankizumab, but not for ixekizumab and guselkumab. Future studies with large groups per drug are required to validate this finding.

AE related drug survival of the first-generation biologics was previously associated with sex, where females showed shorter drug survival than males.^25^ Therefore, we aimed to evaluate AEs using multiple approaches. AEs were more often a decisive reason to discontinue biological therapy for females than males. Using TSQM as a subjective measure of experienced AEs; in the first year of treatment, females reported more often than males to experience AEs from their biologic. Surprisingly, females were not necessarily less satisfied with the safety profile of their biologic. In a previous BioCAPTURE study, the total number of reported AEs was higher in females.^12^ Data from another registry, the Swiss Psoriasis Registry (SDNTT), showed similar results.^26^ However, it is difficult to assess the actual occurrence of the number AEs as a consequence of treatment, because the causal relationship is often difficult to assess, and researchers depend on the reporting of AEs by patients, which can be subjective. Socio-cultural factors may account for the sex-difference in AEs observed in our study. An alternative explanation could be attributed to sex-difference in pharmacokinetics, as higher drug levels and/or longer elimination times in females have previously been shown to be a predictor for AEs for various medications including biologics.^27^ Though, a combination of both explanations cannot be excluded.

Although ineffectiveness related drug survival was lower in females, the course of PASI during the first year of treatment, corrected for confounders, was not different between females and males. The latter suggests an equal effectiveness of treatment for both sexes. This was also confirmed in a recent meta-analysis on clinical characteristics associated with response to biologics, where sex was not associated with treatment response.^28^ What we can tentatively conclude from the contradictory results of the drug survival and PASI analysis, is that females experienced effectiveness of the biologic different form their male counter parts. PASI is assessed by the physician and are therefore an objective measure for effectiveness. Discontinuation of medication due to ineffectiveness (as is presented in drug survival analyses) is more subjective, as it is influenced by how patients experience effectiveness and how much residual disease activity they accept. Our additional finding that females are generally less satisfied with biologic supports the idea of differential experience of medication effectiveness between sexes.

Next to sex, obesity has been associated with shorter drug survival, while PsA was associated with longer drug survival.^29^ In our cohort, the occurrence of PsA but not BMI was statistically significantly different between sexes. Despite the slightly more female patients with PsA than males in our study, biologics were not more often discontinued due to musculoskeletal related AEs in females compared to males.

The strength of this study is that data used for drug survival and other treatment outcomes were extracted from the same prospective registry. The use of GEE to analyze the course of PASI, TSQM and DLQI scores enabled us to longitudinally assess these factors incorporating the different number of treatment episodes per patient and the repeating measurements. Lastly, both drug survival (Cox regression) as GEE analyses were corrected for confounders.

A limitation of this study is that corrected analyses per biologic were not possible due to numbers of patients. In addition, investigating the impact of sex presents a considerable challenge due to the risk for unmeasured confounding. Extracting causal relations about the effects of these outcomes (i.e., PASI, TSQM, DLQI) on the sex-difference in drug survival are difficult to assess due to the observational nature of the study.

This study provides insights in sex-differences for multiple treatment outcomes of biologics, including data of the newer IL17i and IL23i. Biological therapy in general, and specifically for IL17i and IL23i, showed lower drug survival rates for females than males. This might be related to the sex-differences we found for AEs and treatment satisfaction with biologics. In order to improve treatment results in clinical practice for women, further clarification is needed regarding AEs and the reasons why females experience effectiveness of their biologic differently.

## Supplemental Material

Supplemental Material - Lower Drug Survival, Less Satisfaction and More Adverse Events in Females using Biologics for Psoriasis: Results of the Dutch BioCAPTURE Registry Supplemental Material for Lower Drug Survival, Less Satisfaction and More Adverse Events in Females using Biologics for Psoriasis: Results of the Dutch BioCAPTURE Registry by Liana Barenbrug, Renate G. van der Molen, Jake S. F. Maurits, Marieke M. B. Seyger, Marisol E. Otero, Antoni H. Gostynski, Femke M. Homan, Paul M. Ossenkoppele, Inge M. Haeck, Judith. H. J. Hendricksen-Roelofzen, John E. M. Körver, Sharon R. P. Dodemont, Berit Velstra, Maartje A. M. Berends, Lizelotte J. M. T. Weppner-Parren, Romy Keijsers, Annet M. Oostveen, Bas Peters, Roland J. M. Mommers, Martijn B. A. van Doorn, Milan Tjioe, Peter W. Arnold, Astrid L. A. Kuijpers, Marloes M. Kleinpenning, Elke M. G. J. de Jong and Juul M. P. A. van den Reek in Journal of Psoriasis and Psoriatic Arthritis®

## Data Availability

The data that support the findings of this study are not publicly available as participants of this study did not agree for their data to be shared publicly.
