# Supplementary material for: Lower Drug Survival, Less Satisfaction and More Adverse Events in Females Using Biologics for Psoriasis: Results of the Dutch BioCAPTURE Registry
Source: J Psoriasis Psoriatic Arthritis. 2025 Apr 1;10(3):91–100. doi: 10.1177/24755303251327926 (PMC11962928; doi:10.1177/24755303251327926)
Supplement: Supplemental Material - Lower Drug Survival, Less Satisfaction and More Adverse Events in Females using Biologics for Psoriasis: Results of the Dutch BioCAPTURE Registry [file sj-pdf-1-jps-10.1177_24755303251327926.pdf]

Supporting Information:

Table S1| Total number of episodes per biologic included in the study for females and males

Total number of episodes per biologic included in the study	**Total**	**Females**	**Males**	**P**
Adalimumab	568 (30.8%)	223 (28.8%)	345 (32.3%)	0.299
Infliximab	37 (2.0%)	19 (2.5%)	18 (1.7%)	0.142
Etanercept	297 (16.1%)	122 (15.8%)	175 (16.4%)	0.952
Ustekinumab	450 (24.4%)	179 (23.1%)	271 (23.4%)	0.531
Secukinumab	118 (6.4%)	58 (7.5%)	60 (5.6%)	0.064
Ixekizumab	117 (6.3%)	47 (6.1%)	70 (6.5%)	0.844
Brodalumab	38 (2.1%)	18 (2.3%)	20 (1.9%)	0.423
Bimekizumab	8 (0.4%)	5 (0.6%)	3 (0.3%)	0.261
Guselkumab	95 (5.2%)	40 (5.2%)	55 (5.1%)	0.828
Tildrakizumab	15 (0.8%)	7 (0.9%)	8 (0.7%)	0.656
Risankizumab	70 (3.8%)	26 (3.6%)	44 (4.1%)	0.499

Data are expressed as n (%)

Table S2 | Patient and treatment characteristics split per biologic and sex

	**TNFα inhibitors**								
	**Adalimumab**			**Infliximab**			**Etanercept**		
	**Total**	**Females**	**Males**	**Total**	**Females**	**Males**	**Total**	**Females**	**Males**
Number of episodes	568	223	345	37	19	18	296	121	175
Age at onset psoriasis (years; median [IQR])	23.0 [20.0]	21.0 [21.0]	24.5 [19.0]	29.5 [16.1]	25.5 [16.1]	30.5 [17.3]	22.6 [17.0]	20.0 [18.8]	23.0 [16.3]
Disease duration until start of first biologic therapy (years; median [IQR])	18.9 [18.0]	18.5 [21.5]	19.2 17.1]	19.6 [11.1]	19.6 [9.9]	18.5 [12.3]	20.4 [17.2]	20.3 [20.1]	20.7 [15.9]
Age at start of biological therapy (years, mean (SD))	48.9 (13.9)	48.1 (15.0)	49.4 (13.2)	47.1 (13.2)	46.5 (15.4)	47.7 (10.8)	47.3 (12.8)	47.1 (12.7)	47.5 (12.8)
Baseline BMI (kg/m2; median [IQR])	27.6 [7.1]	27.3 [7.2]	27.8 [7.1]	27.2 [12.4]	24.4 [-]	27.2 [18.1]	27.8 [7.1]	28.9 [10.0]	27.7 [4.8]
Baseline PASI score (median [IQR])	9.7 [7.9]	8.3 [8.1]	10.1 [7.5]	14.5 [12.1]	13.4 [14.2]	14.7 [12.6]	12.5 [8.4]	11.8 [9.3]	13.1 [8.2]
PsA (yes)	146 (31.4%)	69 (36.3%)	77 (28%)	25 (67.6%)	13 (68.4%)	12 (66.7%)	88 (32.4%)	38 (34.2%)	50 (31.1%)
Family history with psoriasis (yes)	329 (64.5%)	134 (66.0%)	195 (63.5%)	21 (60.0%)	13 (68.4%)	8 (50.0%)	194 (67.1%)	85 (72.6%)	109 (63.4%)

	**IL17 inhibitors**											
	**Secukinumab**			**Ixekizumab**			**Brodalumab**			**Bimekziumab**		
	**Total**	**Females**	**Males**	**Total**	**Females**	**Males**	**Total**	**Females**	**Males**	**Total**	**Females**	**Males**
Number of episodes	118	58	60	117	47	70	38	18	20	8	5	3
Age at onset psoriasis (years; median [IQR])	23.2 [19.9]	24.0 [27.0]	23.0 [16.5]	24 [20.0]	20.0 [24.7]	27.0 [21.0]	27.7 [22.0]	27.0 [32.0]	28.3 [15.8]	30.0 [30.8]	39.0 [42.0]	27.0 [-]
Disease duration until start of first biologic therapy (years; median [IQR])	22.0 [17.6]	22.4 [20.1]	20.9 [16.0]	22.0 [21.4]	27.0 [22.9]	21.4 [20.1]	22.7 [17.4]	21.3 [24.6]	24.8 [14.3]	16.0 [22.4]	18.8 [28.2]	8.4 [-]
Age at start of biological therapy (years, mean (SD))	50.7 (12.7)	52.9 (14.0)	48.5 (10.9)	51.7 (11.4)	50.1 (12.5)	53.3 (10.5)	77.8 (12.3)	55.3 (14.5)	56.3 (10.2)	56.5 (13.8)	53.0 (10.4)	39.3 (16.6)
Baseline BMI (kg/m2; median [IQR])	26.6 [5.3]	28.1 [6.9]	26.2 [4.1]	28.8 [7.8]	30.9 [12.9]	27.8 [6.0]	26.8 [7.7]	-	26.5 [8.49]	-	-	-
Baseline PASI score (median [IQR])	10.2 [6.8]	7.0 [7.7]	10.7 [8.2]	7.1 [6.7]	6.7 [7.1]	7.1 [6.5]	4.0 [7.1]	6.7 [8.8]	3.9 [6.48]	5.8 [8.0]	3.9 [6.9]	-
PsA (yes)	47 (45.1%)	23 (44.2%)	24 47.1%)	41 (40.6%)	15 (36.6%)	26 (43.3%)	11 (35.5%)	5 (35.7%)	6 (35.3%)	2 (28.6%)	1 (20.0%)	1 (50.0%)
Family history with psoriasis	68	32	36	68	32	36	23	10	13	5	3	2

(yes)	(67.9%)	(58.2%)	(67.9%)	(63.6%)	(72.7%)	(57.1%)	(69.7%)	(62.5%)	(76.5%)	(71.4%)	(60.0%)	(100.0%)
	**IL12/23 and IL23 inhibitors**											
	**Ustekinumab**			**Guselkumab**			**Risankizumab**			**Tildrakizumab**		
	**Total**	**Females**	**Males**	**Total**	**Females**	**Males**	**Total**	**Females**	**Males**	**Total**	**Females**	**Males**
Number of episodes	450	179	271	95	40	55	70	26	44	15	7	8
Age at onset psoriasis (years; median [IQR])	24.0 [22.0]	23.0 [26.5]	24.0 [20.0]	25.0 [21.0]	22.0 [19.5]	25.0 [18.8]	25.0 [23.5]	24.0 [27.9]]	25.0 [17.7]	19.5 [22.0]	32.0 [36.25]	18.0 [10.3]
Disease duration until start of first biologic therapy (years; median [IQR])	18.7 [17.6]	18.4 [17.6]	19.1 [17.3]	21.1 [15.1]	21.1 [23.5]	21.0 [13.0]	22.2 [26.2]	25.4 [28.0]	19.0 [25.2]	29.0 [29.0]	23.5 [30.4]	36.1 [35.6]
Age at start of biological therapy (years, mean (SD))	49.5 (13.6)	49.8 (14.6)	49.2 (13.0)	50.2 (13.6)	50.1 (14.3)	50.2 (13.1)	53.7 (13.7)	57.1 (15.6)	51.7 (12.1)	53.9 (11.9)	57.6 (7.4)	50.7 (14.5)
Baseline BMI (kg/m2; median [IQR])	28.1 [7.1]	28.4 [8.3]	28.0 [6.3]	29.7 [8.4]	28.3 [11.0]	30.2 [7.0]	31.2 [7.1]	28.4 [-]	31.6 [6.5]	28.2 [6.1]	27.8 [-]	28.3 [4.7]
Baseline PASI score (median, [IQR])	11.0 [10.1]	9.6 [10.1]	11.6 [10.3]	8.0 [8.6]	6.6 [7.3]	8.3 [9.8]	7.4 [7.7]	7.5 [11.6]	7.3 [7.0]	7.2 [5.3]	5.1 [4.8]	7.2 [6.3]
PsA (yes)	115 (30.2%)	51 (33.1%)	64 (28.2%)	27 (36.5%)	13 (40.6)	14 (33.3)	19 (31.7%)	9 (36.0%	10 (28.6%)	2 (16.7%)	1 (16.7%)	1 (16.7%)
Family history with psoriasis (yes)	269 (65.3%)	112 (67.9%)	157 (63.6%)	45 (57.0%)	25 (69.4%)	20 (46.5%)	43 (65.2%)	17 (65.4%)	26 (65.0%)	8 (61.5%)	3 (42.9%)	5 (83.3%)

IQR, interquartile range; SD, standard deviation; BMI, body mass index; PASI, Psoriasis Area and Severity Index; PsA, psoriatic arthritis

Appendix S1| Treatment Satisfaction Questionnaire for Medication

Domain Effectiveness:

1. How satisfied you are with the medication's ability to prevent or treat your condition?
   - ☐ Extremely dissatisfied
   - ☐ Very dissatisfied
   - ☐ Dissatisfied
   - ☐ Somewhat satisfied
   - ☐ Satisfied
   - ☐ Very satisfied
   - ☐ Extremely satisfied

2. How satisfied or dissatisfied are you with the way the symptoms are being relieved by this medication?
   - ☐ Extremely dissatisfied
   - ☐ Very dissatisfied
   - ☐ Dissatisfied
   - ☐ Somewhat satisfied
   - ☐ Satisfied
   - ☐ Very satisfied
   - ☐ Extremely satisfied

Domain Side Effects:

3. Do you experience any side effects as a result of using this medication?
   - ☐ Yes
   - ☐ No

4. How dissatisfied are you with the influence of side effects on your physical health and ability to function (e.g., strength, energy levels)?
   - ☐ Extremely dissatisfied
   - ☐ Very dissatisfied
   - ☐ Dissatisfied
   - ☐ Somewhat satisfied
   - ☐ Satisfied
   - ☐ Very satisfied
   - ☐ Extremely satisfied

5. How dissatisfied are you with the influence of side effects on your mental health functioning (e.g., ability to think clearly, stay awake)?
   - ☐ Extremely dissatisfied
   - ☐ Very dissatisfied
   - ☐ Dissatisfied
   - ☐ Somewhat satisfied
   - ☐ Satisfied
   - ☐ Very satisfied
   - ☐ Extremely satisfied

6. How dissatisfied are you with the influence of side effects on your mood or emotions (i.e., fear/anxiety, sadness, irritation/anger)?
   - ☐ Extremely dissatisfied
   - ☐ Very dissatisfied
   - ☐ Dissatisfied
   - ☐ Somewhat satisfied
   - ☐ Satisfied
   - ☐ Very satisfied
   - ☐ Extremely satisfied

Domain Convenience:

7. How satisfied or dissatisfied are you with the ease by which this medication can be administered/used?

- Extremely dissatisfied
- Very dissatisfied
- Dissatisfied
- Somewhat satisfied
- Satisfied
- Very satisfied
- Extremely satisfied

8. How satisfied or dissatisfied are you with the ease by which the time of administration of this medication can be planned?
   - Extremely dissatisfied
   - Very dissatisfied
   - Dissatisfied
   - Somewhat satisfied
   - Satisfied
   - Very satisfied
   - Extremely satisfied

9. How satisfied or dissatisfied are you with how often you need to take this medication?
   - Extremely dissatisfied
   - Very dissatisfied
   - Dissatisfied
   - Somewhat satisfied
   - Satisfied
   - Very satisfied
   - Extremely satisfied

Domain Global Satisfaction:

10. How satisfied or dissatisfied are you with the good aspects of this medication outweighing the bad sides?
    - Extremely dissatisfied
    - Very dissatisfied
    - Dissatisfied
    - Somewhat satisfied
    - Satisfied
    - Very satisfied
    - Extremely satisfied

11. Taking everything into consideration, how satisfied or dissatisfied are you with this one medication?
    - Extremely dissatisfied
    - Very dissatisfied
    - Dissatisfied
    - Somewhat satisfied
    - Satisfied
    - Very satisfied
    - Extremely satisfied

Figure S1| Kaplan Meier curves showing drug survival per type of IL17 or IL23 inhibitor for females and males, split according to reason of discontinuation.

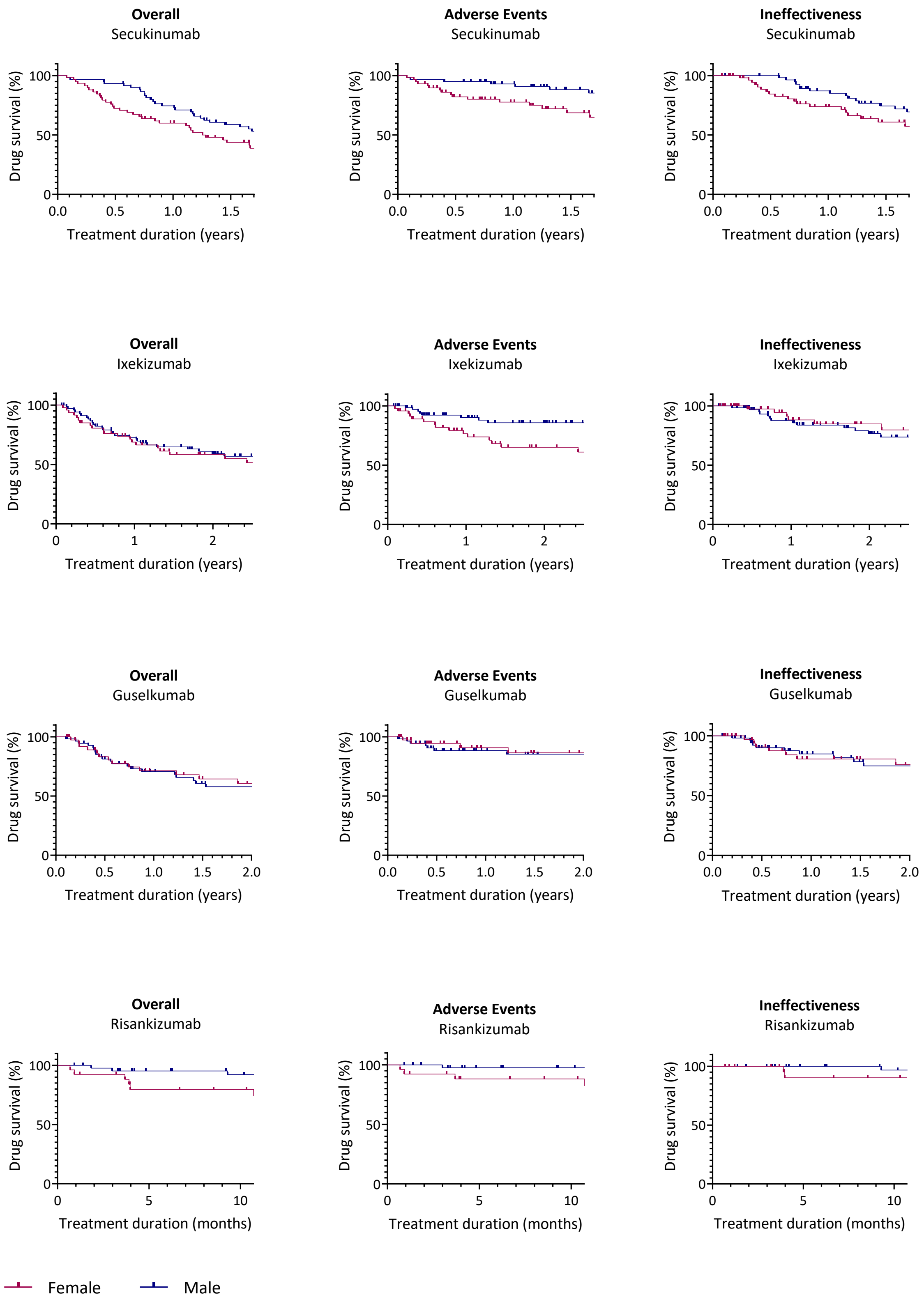

Table S3. Generalized Estimated Equations used to estimate PASI scores during one year follow- up in male (N=879) and female (N=601) psoriasis patients treated with biologics

Variable	Estimate	95% Wald Confidence Interval		p-value [b]
		Lower limit	Upper limit	
Intercept	5.621	4.481	6.760	<0.001
Sex				
Female	0.211	-0.055	0.478	0.120
Male	0[a]	.	.	.
Time from baseline visit				
0	0[a]	.	.	.
3	-6.505	-6.953	-6.058	<0.001
6	-7.230	-7.690	-6.770	<0.001
9	-7.336	-7.801	-6.870	<0.001
12	-7.248	-7.706	-6.789	<0.001
Type of biologic				
Etanercept	0[a]	.	.	.
Adalimumab	-0.359	-0.740	0.22	0.065
Ustekinumab	-0.704	-1.124	-0.284	0.001
Infliximab	-1.221	-3.036	0.594	0.187
Secukinumab	-0.867	-1.404	-0.330	0.002
Ixekizumab	-1.039	-1.633	-0.445	<0.001
Guselkumab	-1.437	-2.156	-0.718	<0.001
Brodalumab	-1.627	-2.455	-0.798	<0.001
Risankizumab	-1.361	-2.001	-0.720	<0.001
Tildrakizumab	-0.66	-1.915	1.783	<0.001
Bimekizumab	-0.098	-0.694	0.498	0.944
Previous use of biologics				
Yes	-0.58	-0.892	-0.265	<0.001
No	0[a]	.	.	.
Psoriatic arthritis				
Yes	0[a]	.	.	.
No	0.050	-0.269	0.370	0.759
Cardiovascular diseases				
Yes	-0.360	-0.730	0.010	0.057
No	0[a]	.	.	.
Diabetes Mellitus				
Yes	0.355	-0.226	0.935	0.232
No	0[a]	.	.	.
IBD				
Yes	-0.491	-1.341	0.359	0.257
No	0[a]	.	.	.
Depression				
Yes	-0.58	-0.587	0.470	0.829
No	0[a]	.	.	.
Baseline PASI	0.354	0.324	0.383	<0.001
BMI	0.058	0.038	0.098	<0.001
Age at start of biologic	0.011	-0.013	0.006	0.462
Disease duration at start of biologic	-0.004	-0.013	0.006	0.462

Abbreviations: IBD, inflammatory bowel disease; PASI, Psoriasis Area and Severity Index; BMI, Body Mass Index
[a] This parameter is set to zero because it is redundant.
[b] P-values associated with type 3 tests of fixed effects.

Table S4. Generalized Estimated Equations used to estimate TSQM Effectiveness scores during one year follow- up in male (N=402) and female (N=271) psoriasis patients treated with biologics

Variable	Estimate	95% Wald Confidence Interval Lower limit	Upper limit	p-value [b]
Intercept	60.068	34.031	86.104	<0.001
Sex				
Female	0[a]	.	.	.
Male	2.136	-0.781	5.053	0.151
Time from baseline visit				
0	-21.932	-25.216	-18.648	<0.001
3	-8.713	-12.388	-5.038	<0.001
6	-2.214	-5.598	1.170	0.200
9	-5.077	-8.412	-1.741	0.003
12	0[a]	.	.	.
Type of biologic				
Etanercept	3.644	-17.468	24.756	0.735
Adalimumab	8.105	-12.771	28.981	0.447
Ustekinumab	9.729	-11.267	30.722	0.364
Infliximab	-19.118	-40.377	2.141	0.078
Secukinumab	8.940	-12.724	30.604	0.419
Ixekizumab	13.383	-8.043	34.809	0.221
Guselkumab	8.438	-13.748	30.624	0.456
Brodalumab	0.151	-21.371	21.673	0.989
Risankizumab	24.409	2.002	46.816	0.033
Tildrakizumab	0[a]	.	.	.
Previous use of biologics				
Yes	0[a]	.	.	.
No	-3.284	-6.178	-0.390	0.026
Psoriatic arthritis				.
Yes	0.748	-2.580	4.076	0.660
No	0[a]	.	.	.
Cardiovascular diseases				
Yes	0[a]	.	.	.
No	-2.175	-7.396	3.045	0.414
Diabetes Mellitus				
Yes	0[a]	.	.	.
No	1.313	-3.446	6.071	0.589
IBD				
Yes	0[a]	.	.	.
No	-2.821	12.531	6.890	0.569
Depression				
Yes	0[a]	.	.	.
No	-2.193	5.640	1.254	0.212
Baseline PASI	0.157	-0.038	0.352	0.115
BMI	0.066	-0.317	0.184	0.604
Age at start of biologic	-0.192	-0.300	-0.084	<0.001
Disease duration at start of biologic	0.078	-0.021	0.176	0.123
TSQM Effectiveness baseline score	0.354	0.219	0.416	<0.001

Abbreviations: TSQM, Treatment Satisfaction Questionnaire for Medication; IBD, Inflammatory Bowel Disease; BMI, Body Mass Index; PASI, Psoriasis Area and Severity Index.
[a] This parameter is set to zero because it is redundant.
[b] P-values associated with type 3 tests of fixed effects.

Table S5. Generalized Estimated Equations used to estimate TSQM Side Effects scores during one year follow- up in male (N=402) and female (N=271) psoriasis patients treated with biologics

Variable	Estimate	95% Wald Confidence Interval		p-value [b]
		Lower limit	Upper limit	
Intercept	49.780	38.359	61.200	<0.001
Sex				
Female	0[a]	.	.	
Male	0.299	-1.304	1.903	0.714
Time from baseline visit				
0	0[a]	.	.	.
3	-1.018	-7.576	-2.944	<0.001
6	-1.037	-4.046	-0.156	0.034
9	-2.101	-2.838	0.765	0.259
12	-5.260	-2.757	0.721	0.251
Type of biologic				
Etanercept	-4.913	-8.192	-1.635	0.003
Adalimumab	-4.867	-7.321	-2.414	<0.001
Ustekinumab	-4.638	-7.303	-1.973	<0.001
Infliximab	-13.069	-17.223	-8.915	<0.001
Secukinumab	-4.210	-7.392	-1.029	0.010
Ixekizumab	-4.207	-7.385	-1.030	0.009
Guselkumab	0.250	-3.193	3.692	0.887
Brodalumab	-7.949	-13.903	-1.994	0.009
Risankizumab	-1.498	-6.678	3.682	0.571
Tildrakizumab	0[a]	.	.	.
Previous use of biologics				
Yes	0[a]	.	.	.
No	-3.154	-4.888	-1.421	<0.001
Psoriatic arthritis				
Yes	-0.770	-2.539	0.999	0.393
No	0[a]	.	.	.
Cardiovascular diseases				
Yes	0[a]	.	.	.
No	-0.416	-2.836	2.005	0.736
Diabetes Mellitus				
Yes	0[a]	.	.	.
No	3.101	-0.010	6.213	0.051
IBD				
Yes	0[a]	.	.	.
No	2.179	-4.552	8.910	0.526
Depression				
Yes	0[a]	.	.	.
No	1.877	-0.708	4.462	0.155
Baseline PASI	0.153	0.057	0.248	0.002
BMI	-0.018	-0.146	0.110	0.785
Age at start of biologic	-0.061	-0.132	0.009	0.089
Disease duration at start of biologic	0.060	-0.003	0.124	0.063
TSQM Side Effects baseline score	0.477	0.421	0.532	<0.001

Abbreviations: TSQM, Treatment Satisfaction Questionnaire for Medication; IBD, Inflammatory Bowel Disease; PASI, Psoriasis Area and Severity Index; BMI, Body Mass Index.

[a] This parameter is set to zero because it is redundant.

[b] P-values associated with type 3 tests of fixed effects.

Table S6. Generalized Estimated Equations used to estimate TSQM Convenience scores during one year follow- up in male (N=402) and female (N=271) psoriasis patients treated with biologics

Variable	Estimate	95% Wald Confidence Interval		p-value [b]
		Lower limit	Upper limit	
Intercept	41.809	17.272	66.347	<0.001
Sex				
Female	0[a]	.	.	.
Male	0.270	-1.656	2.196	0.783
Time from baseline visit				
0	-6.735	-8.912	-4.559	<0.001
3	0.901	-1.402	3.204	0.443
6	-0.567	-2.960	1.827	0.643
9	1.542	-0.734	3.818	0.184
12	0[a]	.	.	.
Type of biologic				
Etanercept	1.167	-19.748	22.082	0.913
Adalimumab	2.076	-18.693	22.844	0.845
Ustekinumab	4.906	-15.938	25.751	0.645
Infliximab	-10.865	-31.867	10.137	0.311
Secukinumab	7.275	-13.737	28.286	0.497
Ixekizumab	4.462	-16.423	25.347	0.675
Guselkumab	6.991	-14.112	28.095	0.516
Brodalumab	-8.775	-30.304	12.755	0.424
Risankizumab	11.237	-10.511	32.985	0.311
Tildrakizumab	0[a]	.	.	.
Previous use of biologics				
Yes	0[a]	.	.	.
No	-1.454	-3.462	0.554	0.156
Psoriatic arthritis				
Yes	-1.980	-4.143	0.183	0.073
No	0[a]	.	.	.
Cardiovascular diseases				
Yes	0[a]	.	.	.
No	2.923	-0.301	6.147	0.076
Diabetes Mellitus				
Yes	0[a]	.	.	.
No	0.116	-3.359	3.590	0.948
IBD				
Yes	0[a]	.	.	.
No	-0.269	-7.449	6.911	0.942
Depression				
Yes	0[a]	.	.	.
No	1.052	1.723	3.827	0.458
Baseline PASI	-0.038	-0.183	0.106	0.604
BMI	-0.127	-0.293	0.040	0.604
Age at start of biologic	-0.030	-0.114	0.056	0.491
Disease duration at start of biologic	0.008	-0.069	0.086	0.831
TSQM Convenience baseline score	0.502	0.436	0.569	<0.001

Abbreviations: TSQM, Treatment Satisfaction Questionnaire for Medication; IBD, Inflammatory Bowel Disease; PASI, Psoriasis Area and Severity Index; BMI, Body Mass Index.
[a] This parameter is set to zero because it is redundant.
[b] P-values associated with type 3 tests of fixed effects.

Table S7. Generalized Estimated Equations used to estimate TSQM Global Satisfaction scores during one year follow- up in male (N=402) and female (N=271) psoriasis patients treated with biologics

Variable	Estimate	95% Wald Confidence Interval		p-value [b]
		Lower limit	Upper limit	
Intercept	46.494	25.277	67.711	<0.001
Sex				
Female	0[a]	.	.	.
Male	2.694	0.515	4.873	0.015
Time from baseline visit				
0	-17.313	-19.933	-14.692	<.001
3	-3.531	-6.125	-.937	.008
6	-1.896	-4.590	.797	.168
9	-0.287	-2.713	2.139	.817
12	0[a]	.	.	.
Type of biologic				
Etanercept	11.809	-5.971	29.588	0.193
Adalimumab	10.733	-6.876	28.341	0.232
Ustekinumab	13.515	-4.172	31.203	0.134
Infliximab	-24.963	-42.872	-7.054	0.006
Secukinumab	13.730	-4.235	31.694	0.134
Ixekizumab	15.332	-2.646	33.310	0.095
Guselkumab	18.026	-0.100	36.151	0.051
Brodalumab	5.213	-13.163	23.589	0.578
Risankizumab	21.055	2.198	39.911	0.029
Tildrakizumab	0[a]	.	.	.
Previous use of biologics				
Yes	0[a]	.	.	.
No	-4.228	-6.549	-1.907	<0.001
Psoriatic arthritis				
Yes	-0.964	-3.228	1.300	0.404
No	0[a]	.	.	.
Cardiovascular diseases				
Yes	0[a]	.	.	.
No	0.352	-3.135	3.840	0.843
Diabetes Mellitus				
Yes	0[a]	.	.	.
No	3.921	-0.243	8.085	0.65
IBD				
Yes	0[a]	.	.	.
No	-1.171	-6.447	4.104	0.663
Depression				
Yes	0[a]	.	.	.
No	1.393	-1.825	4.612	0.396
Baseline PASI	0.051	-0.097	0.198	0.499
BMI	-0.252	-0.434	-0.069	0.007
Age at start of biologic	-0.110	-0.197	-0.022	0.014
Disease duration at start of biologic	0.059	-0.023	0.142	0.159
TSQM Global Satisfaction baseline score	0.427	0.378	0.476	<0.001

Abbreviations: TSQM, Treatment Satisfaction Questionnaire for Medication; IBD, Inflammatory Bowel Disease; PASI, Psoriasis Area and Severity Index; BMI, Body Mass Index.
[a] This parameter is set to zero because it is redundant.
[b] P-values associated with type 3 tests of fixed effects.

Table S8. Generalized Estimated Equations used to estimate DLQI scores during one year follow- up in male (N=428) and female (N=282) psoriasis patients treated with biologics

Variable	Estimate	95% Wald Confidence Interval		p-value [b]
		Lower limit	Upper limit	
Intercept	-2,944	-6.297	0.410	0.085
Sex				
Female	0[a]	.	.	.
Male	-0.121	-0.597	0.354	0.616
Time from baseline visit				
0	6.435	5.754	7,115	<,001
3	0.743	0.207	1,279	0.007
6	-0.101	-0.628	0,426	0.707
9	-0.320	-0.817	0,177	0.207
12	0[a]	.	.	.
Type of biologic				
Etanercept	1.032	-1.236	3.300	0.372
Adalimumab	-0.220	-2.431	1.990	0.845
Ustekinumab	-0.875	-3.092	1.341	0.439
Infliximab	3.075	-4.375	10.525	0.419
Secukinumab	-0.315	-2.816	2.186	0.805
Ixekizumab	-1.398	-3.680	0.884	0.230
Guselkumab	-0.404	-2.894	2.085	0.750
Brodalumab	-0.687	-3.378	2.003	0.617
Risankizumab	-1.192	-3.629	1.246	0.338
Tildrakizumab	0[a]	.		.
Previous use of biologics				
Yes	0[a]	.	.	.
No	1.018	0.518	1.518	<0.001
Psoriatic arthritis				
Yes	-0.007	0.531	0.516	0.978
No	0[a]	.	.	.
Cardiovascular diseases				
Yes	0[a]	.	.	.
No	0.960	0.343	1.577	0.002
Diabetes Mellitus				
Yes	0[a]	.	.	.
No	-0.700	-1.607	0.208	0.131
IBD				
Yes	0[a]	.	.	.
No	1.133	0.298	1.967	0.008
Depression				
Yes	0[a]	.	.	.
No	-0.192	-1.002	0.618	0.131
Baseline PASI	-0.072	-0.101	-0.044	<0.001
BMI	0.050	0.009	0.091	0.017
Age at start of biologic	0.018	-0.003	0.038	0.088
Disease duration at start of biologic	-0.011	-0.026	0.004	0.143
DLQI baseline score	0.424	0.390	0.457	<0.001

Abbreviations: DLQI, Dermatology Life Quality Index; IBD, Inflammatory Bowel Disease; PASI, Psoriasis Area and Severity Index; BMI, Body Mass Index.

[a] This parameter is set to zero because it is redundant.

[b] P-values associated with type 3 tests of fixed effects.
